# Gender Dysphoria and Suicidal Ideation: Clinical Observations from a Psychiatric Emergency Service

**DOI:** 10.7759/cureus.6132

**Published:** 2019-11-12

**Authors:** Derek S Day, John J Saunders, Anu Matorin

**Affiliations:** 1 Medicine, Baylor College of Medicine, Houston, USA; 2 Menninger Department of Psychiatry, Baylor College of Medicine, Houston, USA

**Keywords:** gender dysphoria, suicidal ideation, adolescent, emergency, psychiatry

## Abstract

Adolescent gender dysphoria is increasingly common. There has been documentation of the association of gender dysphoria with numerous other psychiatric conditions as well as attempted and completed suicide. The literature is unsettled on specific risk factors for self-harm within this population. Though there are published recommendations, there appears to be a need for additional clinical evidence for the determination of the safest and most effective treatment strategies for adolescent gender dysphoria.

This clinical observation describes the unique case of an adolescent with gender dysphoria, severe body dysmorphia, and suicidal ideation who presented for emergency psychiatric evaluation. Gender-affirming hormone therapy had been administered to this patient at the age of 13, well earlier than published guidelines, though it was discontinued after a short course due to persistent gender uncertainty and distress. This case provides an opportunity to consider the complexity of adolescent gender dysphoria, including the unique individual features that affect the risk for self-harm and how treatment history may be related. With an increasing prevalence of gender dysphoria in this population, it is essential that every provider who cares for adolescents be well informed and prepared to recognize and respond to these risks.

## Introduction

Gender dysphoria (GD) among adolescents is not uncommon. A recent survey among high school students found that 1.2-2.7% considered themselves transgender or uncertain of gender [1-2]. Reports also suggest an increasing number of adolescents referred to specialized gender identity clinical services for the treatment of gender dysphoria [3]. Any clinician who cares for young patients should be well informed of the impact gender dysphoria and related treatments have on mental health. Awareness of the increased prevalence of depression, anxiety, and suicidal ideation among these individuals can prepare healthcare providers to anticipate and respond appropriately to their presentation.

A 2017 chart review from a transgender clinic in Cincinnati found that among youth aged 12-22 with a diagnosis of gender dysphoria, 58% had another psychiatric diagnosis, and 30.3% reported at least one suicide attempt [4]. Two similar studies support these findings, with attempted suicide rates of 31% and 26% among transgender or gender dysphoric adolescents [2, 5]. New light has been shed on the risk factors for suicidality, including evidence for specific aspects of body dissatisfaction as independent risk factors among GD patients [4]. This case will describe one young patient and discuss these relevant factors.

## Case presentation

A 14-year-old birth-assigned female transitioning to male presented to the emergency department (ED) with a complaint of suicidal ideation. He was reportedly well until 10 years of age when he began to experience depressed mood, body dysmorphia, and gender dysphoria. One year prior to presentation, he established care with outpatient psychiatry and psychology. He had initiated gender-affirming testosterone therapy, but after 30 days of treatment, the patient elected to stop, reporting uncertainty about wanting to be male. He was prescribed aripiprazole 2 mg daily for several months for mood stabilization, reporting a good result; however, aripiprazole was weaned to avoid long-term antipsychotic use, and he was transitioned to fluoxetine 10 mg. No adverse effects were reported.

On the day of their presentation, the patient reported persistent depression, social isolation, worthlessness, and suicidal ideation. His outpatient psychiatrist, who had seen earlier that day, encouraged him to seek emergency evaluation. He stated that he had been experiencing suicidal ideation for over one year, associated with body dysmorphia, gender uncertainty, and the prior process of hormone therapy. He expressed dissatisfaction with height, weight, and appearance, stating, “I am so short. I am so ugly. I am a joke. I look like I am 10 years old. I look stupid. I hate myself. It’s not fair to be me,” and “I don’t want to do anything; I would be better off dead,” but denied any explicit suicidal plan.

The patient had no prior medical or surgical history, including no prior hospital admissions, and took no medications other than fluoxetine as above. On examination, the patient appeared younger than stated age and androgynous, with an unremarkable physical examination otherwise. The mental status examination was unremarkable apart from a dysthymic effect. Vitals, blood count, and electrolytes were within normal limits.

 The patient’s parents were engaged in evaluation and treatment and expressed support of the transition from female to male. They also agreed to voluntary psychiatric admission, which was recommended along with restarting aripiprazole, confirming that his mood had seemed more stable with aripiprazole therapy than fluoxetine alone.

During their stay in the ED, the patient had one emotional outburst after being referred to with female pronouns. He continued to have intermittent suicidal ideation and to frustration with appearance and situation. He tolerated aripiprazole well and agreed to a transfer to an outside psychiatric facility; however, prior to transfer, he developed a sore throat and fever, suspected to be viral in etiology, which prompted a denial of admission. He and his parents expressed a desire to leave immediately at this news, and he was discharged with a 30-day supply of fluoxetine 10 mg and aripiprazole 2 mg and a plan to follow up with his prior psychiatrist.

## Discussion

While the long-term outcome of this patient remains to be seen, this is a significant example of the psychiatric complexity of gender dysphoria. Despite outpatient treatment and supportive family, this patient suffered from persistent suicidal ideation. The repeated expressions of extreme dissatisfaction with appearance were notable. It has been found that among transgender youth, a significantly greater proportion of those who had attempted suicide expressed weight-related body dissatisfaction than those who had not. They also had a higher rate of negative assessment by others of appearance [5]. More recently, another study confirmed a significant correlation between suicidality and a desire for weight change among adolescents with gender dysphoria [4]. There has long been a natural association between body dissatisfaction and gender dysphoria; however, these reports and this case highlight the importance of assessing the degree and characterization of body dissatisfaction as they may contribute to suicidal risk.

Another salient point to consider from this case is the early administration of cross-sex hormone therapy. Treatment guidelines from The Endocrine Society and World Professional Association for Transgender discourage any hormonal treatment prior to Tanner stage 2 and recommend puberty suppression (GnRH) as a reversible first step prior to gender-affirming hormone therapy, which is typically not initiated until age 16 [6-7]. It is likely that a variety of factors played into the decision to forgo pubertal delay in this patient, including the distress attributed to his young appearance relative to peers. However, in his case, gender-affirming therapy also seemed to be associated, by his own description, with increased sadness, confusion, and frustration. De Vries et al. conducted two longitudinal observational studies that assessed the efficacy of recommended therapies and demonstrated improvement in psychological measures after gender suppression, cross-sex therapy at 16, and gender-affirming therapy in adulthood to levels comparable to the general public [8-9]. There is a need for further assessment of the safety and efficacy of the interventions.

## Conclusions

Gender dysphoric patients are at significant risk for psychiatric comorbidities and suicidal ideation and attempts. It is crucial that primary care providers be aware of and diligently evaluate these risks, regardless of treatment status. A collaborative, multi-disciplinary approach can help care for this vulnerable population and avoid tragic outcomes.

## References

[1] Clark TC, Lucassen MF, Bullen P, Denny SJ, Fleming TM, Robinson EM, Rossen FV (2014). The health and well-being of transgender high school students: results from the New Zealand adolescent health survey (Youth '12). J Adolesc Health.

[2] Eisenberg ME, Gower AL, McMorris BJ, Rider GN, Shea G, Coleman E (2017). Risk and protective factors in the lives of transgender/gender nonconforming adolescents. J Adolesc Health.

[3] Aitken M, Steensma TD, Blanchard R (2015). Evidence for an altered sex ratio in clinic‐referred adolescents with gender dysphoria. J Sex Med.

[4] Peterson CM, Matthews A, Copps‐Smith E, Conard LA (2017). Suicidality: self‐harm, and body dissatisfaction in transgender adolescents and emerging adults with gender dysphoria. Suicide Life Threat Behav.

[5] Grossman AH, D'Augelli AR (2007). Transgender youth and life-threatening behaviors. Suicide Life Threat Behav.

[6] Hembree WC, Cohen-Kettenis PT, Gooren L (2017). Endocrine treatment of gender-dysphoric/gender-incongruent persons: an endocrine society clinical practice guideline. J Clin Endocrinol Metab.

[7] Coleman E, Bockting W, Botzer M (2012). Standards of care for the health of transsexual, transgender, and gender-nonconforming people, version 7. Int J Transgend.

[8] de Vries AL, Steensma TD, Doreleijers TAH, Cohen‐Kettenis PT (2011). Puberty suppression in adolescents with gender identity disorder: A prospective Follow‐up study. J Sex Med.

[9] de Vries AL, McGuire JK, Steensma TD, Wagenaar ECF, Doreleijers TAH, Cohen-Kettenis PT (2014). Young adult psychological outcome after puberty suppression and gender reassignment. Pediatrics.

